# Colourimetric Plate Assays Based on Functionalized Gelatine Hydrogel Useful for Various Screening Purposes in Enzymology

**DOI:** 10.3390/ijms24010033

**Published:** 2022-12-20

**Authors:** Karolina Labus, Halina Maniak

**Affiliations:** Department of Micro, Nano and Bioprocess Engineering, Faculty of Chemistry, Wroclaw University of Science and Technology, Wybrzeże Wyspiańskiego 27, 50-370 Wrocław, Poland

**Keywords:** gelatine hydrogel, laccase detection, inhibitor screening, substrate specificity screening, storage stability, microbial cultivation

## Abstract

Hydrogels are intensively investigated biomaterials due to their useful physicochemical and biological properties in bioengineering. In particular, naturally occurring hydrogels are being deployed as carriers for bio-compounds. We used two approaches to develop a plate colourimetric test by immobilising (1) ABTS or (2) laccase from *Trametes versicolor* in the gelatine-based hydrogel. The first system (1) was applied to detect laccase in aqueous samples. We investigated the detection level of the enzyme between 0.05 and 100 µg/mL and pH ranging between 3 and 9; the stability of ABTS in the solution and the immobilised form, as well as the retention functional property of the hydrogel in 4 °C for 30 days. The test can detect laccase within 20 min in the concentration range of 2.5–100 µg/mL; is effective at pH 3–6; preserves high stability and functionality under storage and can be also successfully applied for testing samples from a microbial culture. The second system with the immobilised laccase (2) was tested in terms of substrate specificity (ABTS, syringaldazine, guaiacol) and inhibitor (NaN_3_) screening. ABTS appeared the most proper substrate for laccase with detection sensitivity C_ABTS_ > 0.5 mg/mL. The NaN_3_ tested in the range of 0.5–100 µg/mL showed a distinct inhibition effect in 20 min for 0.5 µg/mL and total inhibition for ≥75 µg/mL.

## 1. Introduction

Currently, a progressive degeneration of the natural environment is being observed due to expansive human activity [1,2,3,4]. In addition to large-scale mining and refining processes, as well as the daily consumption of material goods by humankind, the manufacturing industry has the largest share in the global generation of waste and pollution of all types. In particular, the food, pharmaceutical, and chemical sectors produce millions of tons of by-products, and waste effluents contributing to the high carbon footprint [4,5,6,7,8,9]. Intensive multi-directional research is constantly being carried out to minimise these adverse environmental effects [5,6,10,11]. In this respect, one of the most promising trends is the replacement of classical chemical processes with sustainable biocatalytic transformations [12,13,14,15]. The leading benefit of enzyme use is the possibility of carrying out chemical reactions under mild processing conditions with high selectivity. Moreover, biocatalysts are used not only at the stage of synthesis/conversion of various compounds, but they are also efficient tools for the detection and neutralisation of harmful substances [16,17,18,19]. Hence, it seems reasonable to undertake research in the development of more effective functional biocatalytic systems for various industrial applications. Due to great diversity and relatively easy handling of cultivation, microorganisms are most often used as a rich source of enzymes with valuable catalytic activities, high selectivity, and process stability [20,21,22]. The displacement of polymers and plastics from industrial trade in favour of biomaterials has also a very significant impact on reducing the global formation of nuisance post-production and post-consumer wastes [23,24,25,26,27]. In this case, the main advantage of biomaterials is their easy degradability to environmentally harmless, low-molecular-weight compounds, which can be used as ready-to-use products [27,28,29]. Different types of biopolymers such as polysaccharides or proteins are not only renewable source of valuable mono- and oligomers, but also serve as carriers of bioactive molecules, e.g., drugs, enzymes, agrochemicals, nanoparticles, stem cells, and antibodies [30,31,32,33,34,35,36]. To this extent, more and more attention is being paid to hydrogels. These materials are being intensively researched due to their favourable functional properties, such as biodegradability, biocompatibility, swelling capacity, porosity, semi-permeability, and the ability to create stable multilayer structures [37,38,39]. In particular, they found applications in bioengineering sectors including medicine, pharmacy, tissue engineering, food processing, agriculture, wastewater treatment, environmental protection, biocatalytic processes, and many others [38,39,40,41,42,43,44,45]. Specifically, over the past few years, interest in hydrogels has increased in the context of their use as chemosensors in colourimetric assays. These systems contain an immobilised enzyme or chromogenic/fluorogenic substrate or functional groups in a hydrogel matrix, which react with a target analyte in the sample to give a coloured product. They have been developed mainly as potential diagnostic tools with applications for the detection of molecules, such as ions, low-molecular-weight compounds, and macromolecules. For instance, colourimetric assays were applied for sensing hydrogen [46], nitrite [47,48], nitrate, phosphate [47], and heavy metal ions [49]. In the group of low-molecular-weight compounds, we found cholesterol [50], melanin [51], urea [52], dopamine [53], and bisphenol A [54]. The tests for detecting macromolecules concerned RNA [55] and proteins of specific activity such as streptavidin alkaline phosphatase [56], galactosidase, and glucuronidase [57] or horseradish peroxidase [58]. Both synthetic and natural-originated hydrogel matrices were used in the examples discussed, which are polyalcohols [47,52,54,55,56], polysaccharides [46,49,53,57], and gelatine [44,59,60]. The cited reports do not exhaust the already published colourimetric assays for the detection of specific analytes; however, it should be noted, that only individual examples of the colourimetric research with immobilised laccase in hydrogels were described in the literature so far. In such studies, laccase was used for the reduction of persistent compounds such as bisphenol A [46,47] and synthetic dye [48], but not strictly for detection purposes. Laccase was immobilised on cellulose/alginate composite hydrogel [46], in poly(ethylene glycol) [47], or alginate/gelatine hydrogel [48]. The other usage of the colourimetric test was the entrapment of a substrate in the solid agar medium and used for identifying the microbes producing laccase [49,50]. Apart from immobilisation in hydrogel matrices, another example was the application of the ABTS-impregnated paper discs for the determination of laccase activity. Such a non-hydrogel assay was applied during the purification of this enzyme on chromatographic columns [51], but practical application, possibilities, and limitations have not been presented in detail.

The system we propose is also based on the use of a substrate that in the presence of a particular enzyme undergoes visible conversion to a coloured product. However, we show the broader applicability of such a system in two opposite modes. In our approach, one of the bioactive compounds (depending on the application: substrate or enzyme) was immobilised within the naturally originated hydrogel matrix to obtain the stable testing kit. For this purpose, we used gelatine, which is a readily available, cheap compound obtained as a side product of commercial animal and fish processing [27,52,53]. Importantly, it is certified by U.S. Food and Drug Administration as a GRAS compound (Generally Recognised As Safe) [54] and is commonly used as a gelling agent for food [27,52,55,56], cosmetics [57,58], medicines [39,56,58,59], pharmaceuticals [54,56,58], and in other branches [39,60]. Furthermore, gelatine has favourable physicochemical properties as a hydrogel matrix. It provides suitable reaction conditions for enzyme activity and is easily biodegradable to non-toxic, low-molecular-weight compounds [36,39,43,44,61]. The selection of gelatine was additionally supported by the results obtained in our previous work, where the properties of alginate and gelatine hydrogel as enzyme carriers were compared [62]. In this case, the gelatine-based hydrogel enzymatically cross-linked with microbial transglutaminase (mTGase) was a more efficient support for the permanent immobilisation of the given enzyme.

We would like to underline that our concept of a colourimetric detection test based on hydrogel matrices enriched with bioactive ingredients can be applied to enzymes with various catalytic activities. We have already started considering the development of such a solution for selected enzymes in previous research for *β*-galactosidase [63,64], and tyrosinase [65]. However, it was only applied in the case of detecting their presence in the tested samples, whereas in the current study, we would like to present the multifunctionality of using gelatine hydrogel matrices containing targeted active compounds in enzymological screening studies. To precisely analyse the possibilities of the practical application of the provided test, we used the laccase (EC 1.10.3.2) as a model enzyme, and its substrate, 2,2′-azino-bis(3-ethylbenzothiazoline-6- sulfonate) sodium salt (ABTS). 

## 2. Results and Discussion

**Figure 1:**
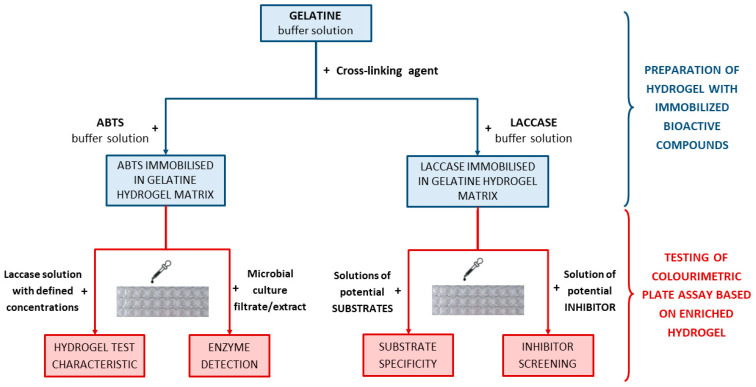
Scheme of research on the development of hydrogel-based colourimetric tests for various screening purposes in enzymology using laccase as the model biocatalyst.

For convenience and readability of the experiments undertaken, we have schematically presented the particular stages of the research considered (Figure 1). Briefly, the first stage, highlighted in blue, involves the preparation of the hydrogel matrices enriched with (1) ABTS (left side) and (2) laccase (right side). The second step, marked in red, includes the example tests and applications of both types of hydrogel matrices developed in the current study.

### 2.1. Hydrogel-Based Test for Colourimetric Detection of Laccase

A quick and simple test that allows the effective detection of a biocatalyst with a specific activity is a desirable analytical tool to improve the preliminary enzymatic screening in both microbiological cultures and other multicomponent mixtures.

In our research, we focused on preparing a diagnostic test which was based on gelatine hydrogel matrices enriched with a substrate (2,2′-azino-bis(3-ethylbenzothiazoline-6-sulfonate) sodium salt, ABTS) of laccase for the detection of its activity in a sample. The following functional parameters for detection sensitivity, operating pH range, and storage stability were determined. Validation of the proposed solution was performed using a commercial laccase preparation from *Trametes versicolor*. In the next stage, the suitability of the developed assay for screening laccase in culture fluids during the cultivation of *Cerrena unicolor* was tested.

#### 2.1.1. Development of the Test for the Detection of Laccase in Aqueous Solutions

**Figure 2:**
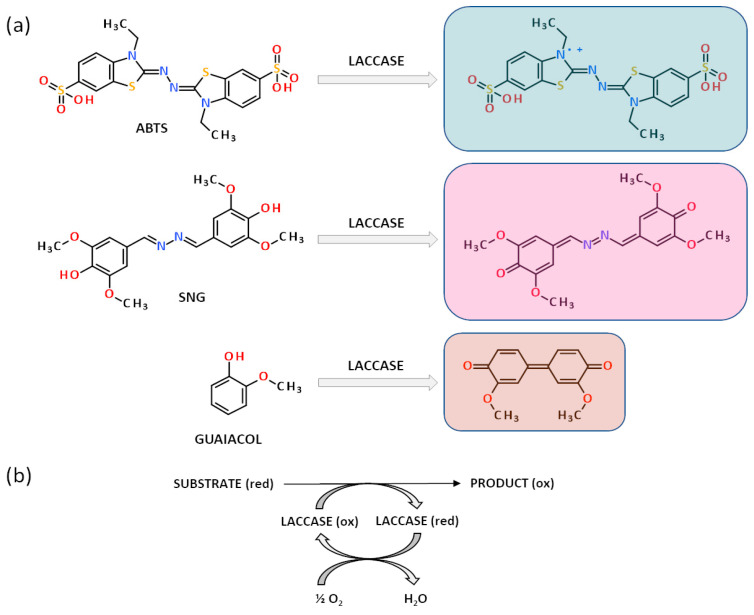
Laccase catalysed reactions with typical synthetic substrates. (**a**). The oxidation of ABTS (upper), syringaldazine (SNG, middle), and guaiacol (lower) to colourful products: green-blue, pink-violet, and orange-brown, respectively. (**b**). The scheme of substrate oxidation by laccase is part of the red-ox reaction where the enzyme undergoes reduction by substrate oxidation and in turn, it returns to its native oxidised form by transferring the electrons to the oxygen molecule which is a final electron acceptor.

The typical substrates used for measurements of laccase activity were presented in Figure 2. The colourimetric test for laccase detection proposed in this study was based on gelatine hydrogel matrices enriched with 2,2′-azino-bis(3-ethylbenzothiazoline-6-sulfonate) sodium salt (ABTS)—one of the well-known synthetic substrate of this enzyme [49,51,66,67,68,69]. Syringaldazine (SNG) [70,71,72,73,74,75] and guaiacol [49,50,67,76] are other substrates frequently used in the determination of laccase activity. Unfortunately, their application in hydrogel-based tests has some limitations which were discussed in Section 2.2.1. In the assay with ABTS, the presence of laccase was indicated by the appearance of a green-blue product resulting from the biocatalytic oxidation of ABTS to its radical cation (Figure 2b).

**Figure 3:**
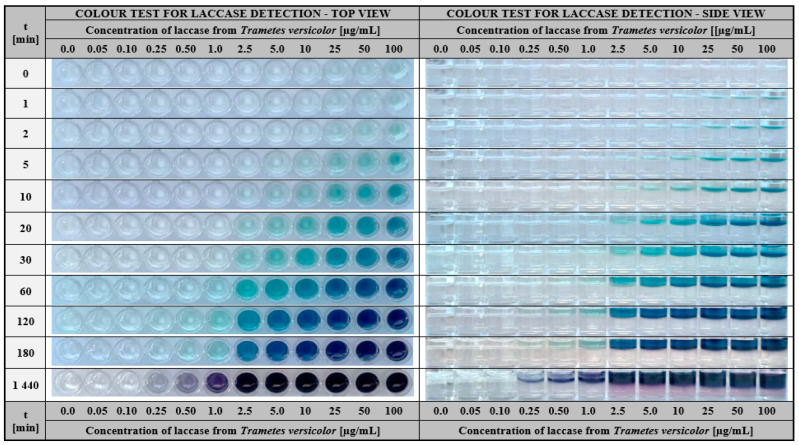
The visual response obtained after a different time (0–1440 min) of hydrogel-based test with immobilised 1.5 mg/mL ABTS performed for laccase from *Trametes versicolor* solutions with a concentration range of 0.05–100 µg/mL, left—top view, right—side view.

The proposed colourimetric assay for visual detection of laccase is based on the use of ABTS entrapped in gelatine-based hydrogel introduced into a 96-well plate. The methodology proceeds as follows: a small amount of aqueous solution potentially containing this enzyme was applied to the surface of the ABTS-enriched hydrogel matrix and the colour change from transparent to green-blue was monitored over time. The appearance of colour indicated the presence of laccase in the test solution. Whereas, the intensity of the colour that appeared after a defined time allowed us to estimate the approximate enzyme concentration. Applying this procedure, we performed detailed studies to determine the functional properties of the proposed diagnostic test. Firstly, we have taken into consideration its detection sensitivity. For this purpose, the laccase solutions of different concentrations (0.05–100 µg/mL) were prepared and added on the surface of gelatine hydrogel matrices, as shown in Figure 3.

As early as 5 min after the samples were applied, a distinct change in the gel colour was observed for laccase in the concentration between 10 and 100 µg/mL (Figure 3, side view). Prolonging the assay time to at least 20 min increased the sensitivity of the test which enabled the detection of the laccase concentration even at 2.5 µg/mL. Further extension of the time to 120 min resulted in a colour response in the ABTS test for two successively lower concentrations of this enzyme (1.0 and 0.5 µg/mL). Moreover, the results after 24 h (Figure 3, last row), revealed the presence of laccase activity as low as 0.25 µg/mL. Concluding this part of the research, the satisfactory results of visual laccase detection in the test are obtained for enzyme concentrations between 2.5 and 100 µg/mL and higher. In this case, the required duration of the analysis is at least 20 min. Nevertheless, it is also possible to detect lower concentrations of laccase (starting from 0.25 µg/mL), but then the test time should be extended to 24 h.

**Figure 4:**
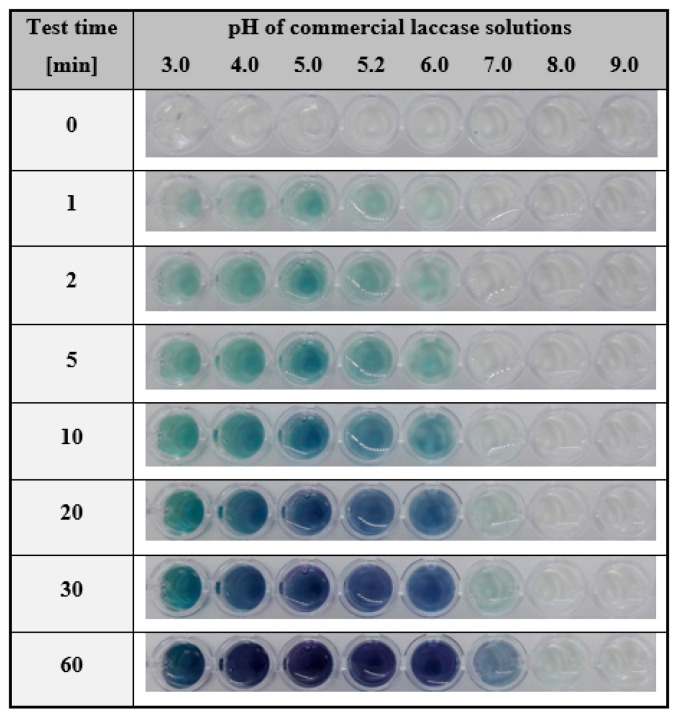
Visual response of the assays in a hydrogel matrix with immobilised ABTS (1.5 mg/mL) obtained after exposure time between 0 and 60 min for laccase solution (100 µg/mL) prepared in the citrate-phosphate buffer with pH ranging from 3.0 to 9.0.

Based on the literature review, laccases of different origins may have a varying range of preferential pH [77,78]. Therefore, in the next step, we verified the possibility of detecting this enzyme under different pH conditions. For that purpose, we used the hydrogel with immobilised ABTS and laccase solutions in one fixed concentration, prepared in buffers at pH ranging from 3.0 to 9.0. Such samples were subjected to colourimetric detection according to the developed procedure. The results of the experiment are shown in Figure 4.

As could be observed after just 2 min, the change in gel colour became rapidly visible for enzyme solutions at a pH from 3.0 to 6.0. Prolongation of the analysis time resulted in a deepening blue-green colour in this pH range, and 60 min was required to visualise the presence of the laccase activity at pH 7.0. Based on these results, it was concluded that the activity of commercial laccase from *Trametes versicolor* in the proposed assay conditions certainly could not be determined at pH 8.0 and above; however, one should take into account that the test results reflected the pH range at which the enzyme exhibited activity. In fact, the pH range at which the laccase from *T. versicolor* preserves the ability to oxidise ABTS is between 3 and 6 [79,80] with the optimum falling at around pH 5 [69,80,81]. Thus, this colourimetric test is also a suitable tool for qualitative diagnosis of the spectrum of enzyme activity, depending on the pH applied.

**Figure 5:**
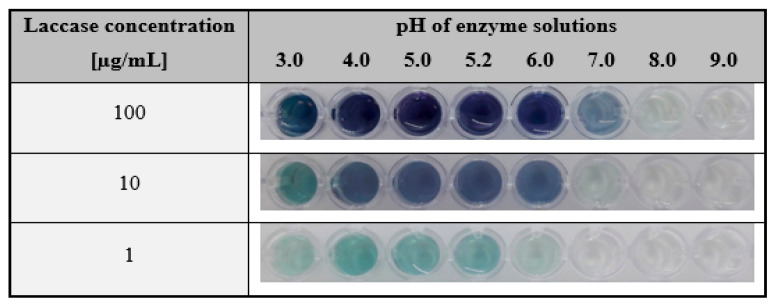
The results of the colourimetric test for the different concentrations of laccase in phosphate-citrate buffer with fixed pH values. The comparison of the detection level of enzymatic activity was examined for laccase concentrations of 100, 10, and 1 µg/mL and in the pH range of 3.0–9.0. Test conditions: hydrogel gelatine matrices containing ABTS at a concentration of 1.5 mg/mL; test time: 60 min.

The next step in the development of the hydrogel test with immobilised ABTS was to determine the sensibility of laccase detection at different pH values. The enzyme was prepared in the concentration range of 1–100 µg/mL and fixed pH values between 3 and 9. As shown in Figure 5, regardless of the concentration used, the presence of the colour was observed in the pH between 4.0 and 5.2. At the lowest enzyme concentration (1 µg/mL), the effect of ABTS oxidation was also detected at pH 3.0 and 6.0, but the colour of the product was visible at the perceptual border. As mentioned above, these pH values overlapped the pH range at which laccase from *T. versicolor* preserves the ABTS oxidation activity [79,80].

To summarise this part of the research, it may be concluded that the proposed colourimetric assay based on immobilised ABTS in a gelatine matrix was effective in detecting laccase at its relatively low concentrations. A key aspect was the pH of the tested solutions. The values of pH should coincide with the pH range of the enzyme’s catalytic activity. In the case of the commercial enzyme used here, the level of detection of laccase activity was observed even at its lowest concentration, i.e., 1 µg/mL, in the pH range of 3–6.

**Figure 6:**
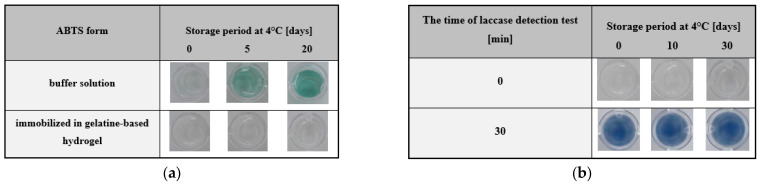
Evaluation of storage stability for ABTS—a substrate used for determination of laccase activity in a gelatine hydrogel-based colourimetric test. ABTS was tested in its native form in a phosphate-citrate buffer solution at pH 5.2 (**a**), and as immobilised in a hydrogel matrix after 0–20 days (**b**). Storage stability of gelatine hydrogel test with immobilised ABTS was performed after 30 days. Laccase detection assays were compared after 30 min of colourimetric test applying enzyme concentration of 100 µg/mL. Experimental conditions: 4 °C, and with 1.5 mg/mL of ABTS.

A very important functional parameter that determines the commercial applicability of any type of chemical or enzymatic test is its storage stability. An economically desirable characteristic is the longest possible shelf life of the proposed product. Therefore, our next experiments were concerned with the determination of the storage stability of both the substrate (ABTS) chosen to measure enzymatic activity as well as the ABTS-containing gelatine hydrogel matrix. The ABTS was examined in the form of a buffer solution and immobilised in a hydrogel matrix. The results of ABTS storage in particular form was shown in Figure 6a. The buffer solution of ABTS underwent slow but visible auto-oxidation after only 5 days of storage at 4 °C. While the ABTS entrapped in the gelatine hydrogel was stable for 20 days of storage under similar conditions.

When considering the storage stability of the entire system tested, a similar procedure was applied. The gelatine matrices containing ABTS were left in closed well plates at 4 °C for 30 days. During this time, the determination of storage stability was performed for the hydrogel matrix at days 10 and 30 and for the matrix at the start of the test (control). The results of the colourimetric response of the test for time t = 0 min and after 30 min for the storage period considered were presented in Figure 6b. A visual comparison of the results of the laccase detection test indicated that there were no differences in the response of the system, and the colour intensity for the storage times (day 0, 10, and 30) was comparable. In conclusion, the experimental results justified that immobilisation of the substrate in the hydrogel matrix as a key step in the development of a colourimetric test. Furthermore, the developed hydrogel-based assay was stable throughout the storage period, which confirms the validity of the approach considered in the current study. This approach provided suitable conditions for the determination of laccase activity and ensured the long-term stability of the developed test.

**Figure 7:**
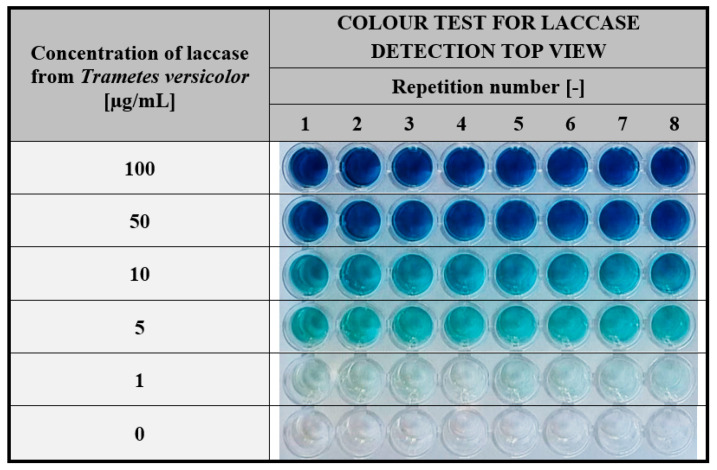
Repeatability of the visual responses of the hydrogel-based test performed for the different concentrations of laccase in phosphate-citrate buffer pH 5.2. Test conditions: hydrogel gelatine matrices containing ABTS at a concentration of 1.5 mg/mL; test time: 60 min.

Another important parameter defining the reliability of the proposed colourimetric assay is the repeatability of the detection response obtained for a given concentration of the enzyme. As shown in Figure 7, the colour intensity is visually comparable in all 8 replicates performed for each of the tested solutions.

These results demonstrate that the test developed in our study is reliable over the entire range of laccase concentrations used (1–100 µg/mL) and can be applied effectively for various screening research in enzymology.

#### 2.1.2. Application of the Hydrogel-Based Colourimetric Assay in Microbiological Cultures

Microbiological cultures are long-term processes usually occupying several to dozens of days. During this time, studies are conducted on the growth of the microorganism, the products released and the substrate consumed. Analytical methods used in monitoring changes in microbial cultures require special equipment, reagents, and specific physico-chemical conditions. Samples for these analyses often require dilution to contain a specific concentration of an analyte. Some analyses take several hours (determination of dry weight), while others require elevated temperatures or specialised reagents and developed standard curves. Therefore, any opportunity to reduce the duration of sample preparation and analysis is highly desirable.

**Figure 8:**
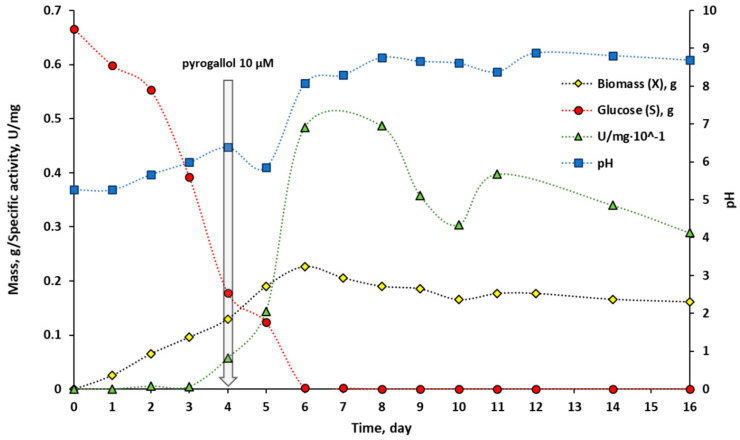
The course of *Cerrena unicolor* cultivation. The legend: yellow diamond—fungal dry mass [g], red circle—glucose (substrate) mass [g], green triangle—specific laccase activity [U/mg], blue square—pH in culture medium. The set of similar points was linked with a dotted line for clarity. The starting glucose mass was 0.63 g, on the 4th day the culture was induced with 10 µM of pyrogallol.

To verify the applicability of a quick test based on the colour reaction of the investigated sample with the substrate entrapped in the hydrogel matrix, a microbial cultivation experiment was planned. We employed a fungus *Cerrena unicolor* that produces an extracellular laccase secreted into the culture medium. The detailed characteristics and process parameters that resulted from microbiological culture may be found in Supporting Materials Table S1. The general assumption of the experiment was to quantitatively analyse laccase activity by spectrophotometric measurement of the ABTS oxidation rate expressed in U/mg units and compare it with the analytical results obtained in hydrogel assays. *Cerrena unicolor* cultivation results were presented in Figure 8. Briefly, the monitoring of changes in biomass (X), glucose (S), specific laccase activity (U/mg of protein), and pH was conducted for 16 days.

**Figure 9:**
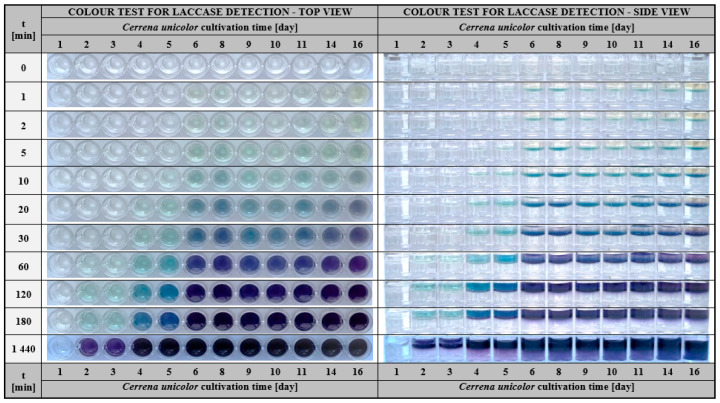
The visual response of colourimetric assay measurements performed for *Cerrena unicolor* culture samples (day 1–16) measured at different times (0–1440 min) using a hydrogel-based assay with immobilised ABTS (1.5 mg/mL); left—top view, right—side view.

Spectroscopic measurements reflecting the course of laccase activity (green triangles) showed that the production of the enzyme appeared on day 4 of culture and increased rapidly reaching a maximum on days between 6 and 8. The analysis of the course for the specific laccase activity revealed another peak of maximum activity recorded on day 11. This phenomenon is known and typical for the white-rot fungi cultures and could be attributed to the production of laccase isoforms at different stages of growth [82,83,84]. After day 11, a decrease in specific laccase activity was observed, associated with substrate depletion and culture collapse. Figure 9 showed the results of a colourimetric hydrogel-based test performed for analogous samples measured spectrophotometrically.

Determination of laccase activity with a colourimetric test allowed detection of the enzyme activity as early as 5 min after the sample was applied on the hydrogel surface. At this time, it was possible to determine the samples with the most concentrated active protein that was for samples corresponding to days 6–16 (Figure 9, side view). At the 20 min of the test, the substantial effect was visible on days 6–8 and 11. These visual response corresponded to the maxima of laccase activity determined spectrophotometrically (Figure 8) and were reflected by the more intense colour of the oxidised ABTS in the hydrogel matrix. After 2 and 3 h, it was possible to detect even the smallest amount of laccase in samples corresponding to the initial days of culture—days 2 and 3. Such determinations were not observed in the spectrophotometric measurement of enzyme activity. Based on the results, it appeared that laccase from *Cerrena unicolor* could be detected with the hydrogel test even in a culture solution having a slightly alkaline pH, such as pH 8.1–8.8 (Figure 8, blue square). A comparison of the above results with a commercial preparation, for which detection was possible up to pH 6 (Figure 5), showed that pH is an important factor for determining enzyme activity in samples of different origins. Laccases from *C. unicolor* show higher pH tolerance [82,83], when compared to laccase from *T. versicolor*, since *C. unicolor* production medium, reaches the alkaline pH [85] and therefore laccase activity could be detected by the colourimetric test.

To sum up the results of the experiment, it should be claimed that assays based on the determination of laccase activity in a gelatin hydrogel matrix enriched with ABTS are tests ready to use since they do not require the preparation of any additional reagents and specific reaction conditions. An important advantage of the test is the size of the sample introduced on the surface of the matrix, which is in the range of 50–250 µL, this preserves a significant amount of material for testing and performing other additional analyses. Furthermore, a distinctive feature of this assay is the rapid response in the hydrogel matrix, which becomes visible in the form of a coloured stripe, ensuring ease of reading. The intensity of this colour increases over time and enables one to conclude which sample contains significant amounts of the enzyme in a relatively short time (in 5 min). Finally, the analyses are performed at room temperature so they do not require the application of any additional equipment.

### 2.2. Gelatine Hydrogels Containing Immobilised Laccase for Various Purposes in Enzymology

Following the success achieved in the case of developing a detection test for laccase using ABTS-enriched gelatine matrices, it was decided to use the potential of such enzyme/substrate/support system in a reversed mode. In this approach, the main functional element of the test was laccase immobilised by entrapment in a gelatine-based hydrogel. In this approach, such type of assay was examined in terms of suitability for screening potential substrates and inhibitors of the tested enzyme.

#### 2.2.1. Hydrogel-Based Assay for Colourimetric Screening a Substrate Specificity of Laccase

**Figure 10:**
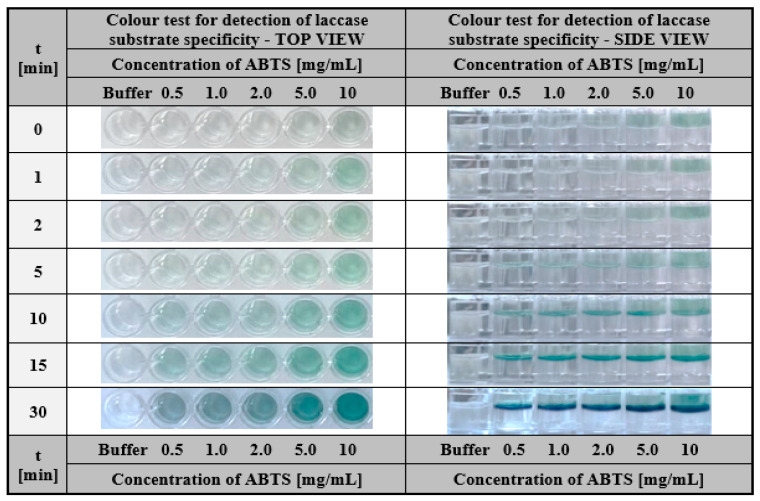
The visual response obtained after a different time of hydrogel-based test with immobilised laccase from *Trametes versicolor* (final concentration 200 µg/mL) was performed for ABTS solutions with a concentration in the range of 0.5–10 mg/mL.

**Figure 11:**
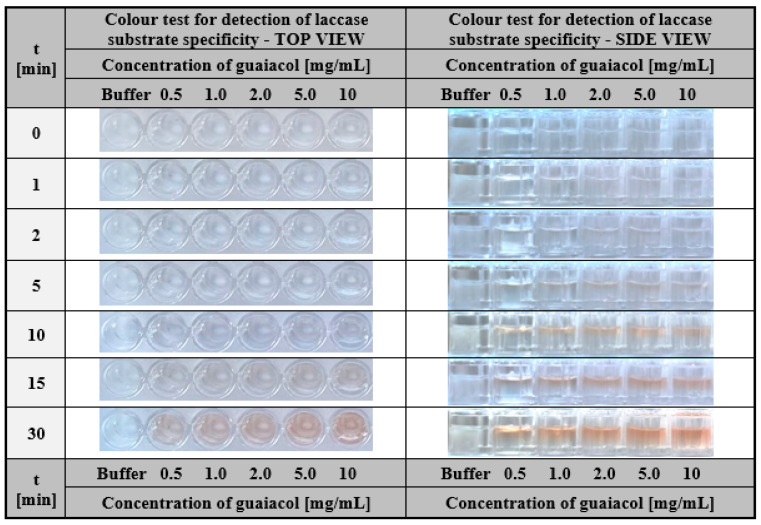
The visual response obtained after a different time of hydrogel-based test with immobilised laccase from *Trametes versicolor* (final concentration 200 µg/mL) performed for guaiacol solutions with a concentration in the range of 0.5–10 mg/mL.

**Figure 12:**
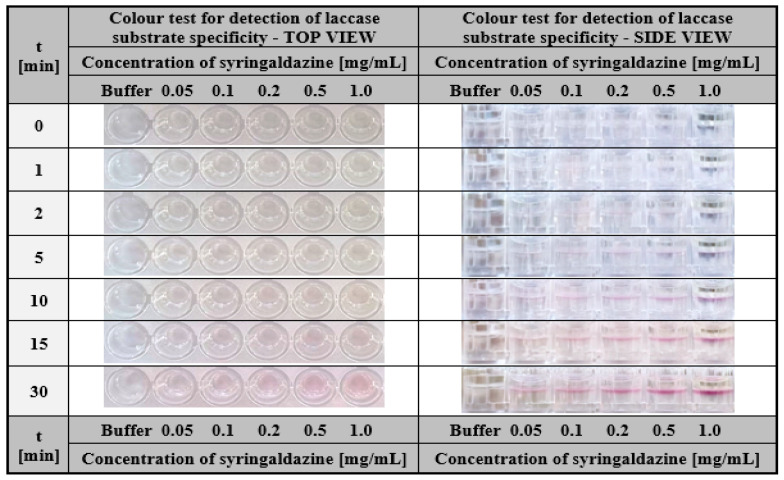
The visual response obtained after a different time of hydrogel-based test with immobilised laccase from *Trametes versicolor* (final concentration 200 µg/mL) performed for syringaldazine solutions with a concentration in the range of 0.05–1.0 mg/mL.

Laccase-enriched gelatine matrices served as a colourimetric assay to determine the substrate specificity of this enzyme. To demonstrate the application potential of laccase-enriched gelatine matrices for substrate testing, we used three compounds most commonly used to determine the activity of this enzyme—ABTS, syringalazine (SNG) and guaiacol. As mentioned earlier, their common feature is that they are all oxidised into coloured products by laccase (Figure 2a,b), which is essential for an effective visual response in the assay. The tests were conducted for different concentrations of each compound. For ABTS and guaiacol, the range was 0.5–10 mg/mL. Whereas, due to the low solubility of syringaldazine, the concentrations were one order lower (0.05–1.0 mg/mL). Colourimetric results obtained after different test times for ABTS, guaiacol, and SNG were depicted in Figure 10, Figure 11 and Figure 12 respectively.

One can notice that in all cases, a positive response was visible in the form of a colour stripe after only 10 min for all the solutions used; however, in the case of ABTS, the colour change for each concentration was the most apparent (Figure 10). This was one of the main reasons for using ABTS as a reference substrate in all the studies presented in this paper. The obtained results indicate the high potential of laccase-enriched gelatine hydrogel matrices as the screening test for substrates of a given enzyme. We would like to emphasise that also other enzyme-substrate systems can be analysed using this concept of the detection assay. There are only two restrictions: (i) the enzyme should retain its properties after immobilisation in the hydrogel matrix and (ii) the analysed compounds should be converted into coloured products upon contact with the biocatalyst.

#### 2.2.2. Hydrogel-Based Assay for Colourimetric Screening Potential Inhibitors of Laccase

**Figure 13:**
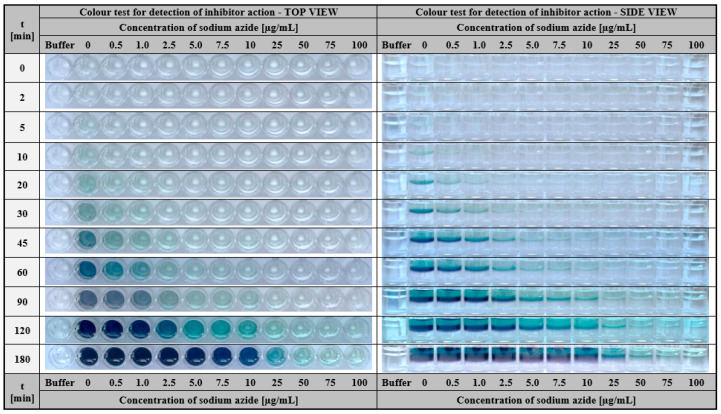
The visual response obtained after a different time of hydrogel-based test with immobilised laccase from *Trametes versicolor* (final concentration 200 µg/mL) performed for a fixed concentration of ABTS (1.5 mg/mL) containing different concentrations of sodium azide (0.5–100 µg/mL).

The next possible application of gelatine matrices containing immobilised laccase as a bioactive agent is screening potential inhibitors of this enzyme. Studies on the identification of effective enzyme inhibitors are particularly time-consuming and require a considerable number of experiments and analyses [74,75]. Preliminary studies require the testing of a large group of compounds in different concentrations. Therefore the use of a rapid colourimetric assay with immobilised enzyme significantly reduces the time of prescreening and allows the selection of potentially active inhibitors for further kinetic studies in a short time. In order to demonstrate the feasibility of such test in practice, sodium azide (NaN_3_) was used as a well-known laccase inorganic inhibitor. In the study, a colourimetric assay was performed using a fixed concentration ABTS mixture (1.5 mg/mL) and various concentrations of sodium azide (0.5–100 µg/mL). The potency of this inhibitor was investigated by observing the colour change of individual samples over time with relation to a reference sample containing only the substrate solution (ABTS). As expected, as the concentration of NaN_3_ increased, the intensity of the green-blue colour decreased (Figure 13). This means that by applying the colourimetric assay presented here, it is possible not only to effectively screen potential laccase inhibitors, but also to preliminarily estimate the concentration of this compound that rapidly inhibits catalytic activity.

### 2.3. Results Discussion

Current research has resulted in the development of multifunctional colorimetric assays based on ABTS or laccase immobilised in gelatine hydrogel matrices. Through the use of this biopolymer support enriched with bioactive compounds, long-lasting, sensitive test kits for various enzymological screening studies were provided. In our study, we proposed two approaches for this enzyme/substrate/hydrogel system. The first one uses hydrogel matrix containing ABTS, which enables the sensitive detection of laccase in a relatively short time (20 min) in a wide range of concentrations (2.5–100 µg/mL) and pH (3.0–6.0). While the second is based on the reverse mode (laccase immobilised in a gelatine support) and enables effective screening for substrates already within 10 min (ABTS and guaiacol in the range of 0.5–10 mg/mL; syringaldazyne 0.05–1.0 mg/mL) and potential inhibitors within 30 min (sodium azide 0.5–100 µg/mL). To the best of our knowledge, the solutions demonstrating the versatility of using the hydrogel/laccase/ABTS system for the development of rapid visual screening tests proposed in our study are novel and not described in detail in the available literature. In previous reports, one can only find examples of using hydrogel-immobilised laccase for the removal of various compounds from aquous solutions (e.g., bisphenol A [46,47], synthetic dye [48]). In turn, the use of ABTS retained on some support for the detection of laccase has been described only for paper discs impregnated with this compound [51], and due to incomplete data in the source article (laccase concentration in the tested samples was not given), the results cannot be directly compared with each other.

## 3. Materials and Methods

### 3.1. Materials

Potato dextrose agar was purchased from Merck (Warsaw, Poland), glucose test was from Biomaxima (Lublin, Poland), 2,2′-azino-bis(3-ethylbenzothiazoline-6-sulfonate) sodium salt (ABTS), laccase from *Trametes versicolor* (EC 1.10.3.2), bovine serum albumin, porcine skin gelatine, sodium azide (NaN_3_) and Lowry reagent were from Sigma-Aldrich (Poznan, Poland). Other chemicals were purchased from POCh (Gliwice, Poland). Transglutaminase (mTGase) Activa^®^WM was kindly donated by Ajinomoto (Tokyo, Japan). All the chemicals used were of analytical grade.

### 3.2. Methods

#### 3.2.1. Preparation of Gelatine Hydrogel Matrices Containing Reactive Compounds

Gelatine hydrogel matrices with immobilised 2,2′-azino-bis(3-ethylbenzothiazoline-6- sulfonate) sodium salt (ABTS) or a commercial preparation of laccase from *Trametes versicolor* were prepared according to a modified procedure described in the previous study [86]. Briefly, a weighted portion of gelatine (15% *w*/*v*) was dissolved in 0.1 M citrate-phosphate buffer (pH 5.2) in a glass beaker thermostated at 80 °C for approximately 30 min. Next, the solution was cooled to 45 °C and incubated at this temperature for a few minutes. In parallel, a buffer solution of ABTS or laccase with given concentration was prepared. Then, after total dissolution, a weighted portion of cross-linking agent (microbial transglutaminase, mTGase) was added to obtain its concentration of 3% *w/v*. The cross-linking step began by mixing the ABTS/mTGase or laccase/mTGase solution with gelatine at the volume ratio of 1:2. The blend thus obtained was pipetted into the 96-well plate (85.4 × 127.6 × 14.4 mm) with 200 µL for each hole (diameter: 6.5 mm; height: 10.8 mm) and stored until use at 4 °C. The gelatine hydrogel and the 96-well plate used are shown in Figure S1. In our study, concentrations of 4.5 mg/mL ABTS and 600 µg/mL commercial preparation of laccase were used for immobilisation purposes.

#### 3.2.2. Hydrogel-Based Assay for Colourimetric Detection of Laccase

The test for laccase detection was based on the monitoring of the colour change of the gelatin matrix containing the immobilised ABTS from transparent to green-blue. The appearance of the colour indicates the presence of laccase in the tested sample, and the intensity of the green-blue colour enables us to estimate the concentration of laccase.

The test was performed by applying 100 µL of an aqueous solution potentially containing laccase to the surface of a hydrogel containing ABTS placed on a microlitre well plate with a volume of 200 µL and monitoring the appearance of the colour in time from transparent to green-blue, or lack thereof.

#### 3.2.3. Determination of Laccase Concentration Range Effectively Detected by the Hydrogel-Based Assay

First, solutions of laccase from *Trametes versicolor* with various concentrations (0.05–100 µg/mL) were prepared in 0.1 M citrate-phosphate buffer (pH 5.2). The protein concentrations in the enzyme solutions were determined by using the Lowry method [87]. Then, a 100 µL of each laccase preparation was applied to the gelatine-based hydrogel matrix containing ABTS, and the progress of the change in gel colour from transparent to green-blue was monitored in time.

#### 3.2.4. Determination of the pH Range in which Laccase Is Effectively Detected by the Hydrogel-Based Assay

First, solutions of laccase from *Trametes versicolor* in a given concentration (200 µg/mL) were prepared in 0.1 M citrate-phosphate buffer with different pH (range of 3.0–9.0) Then, a 100 µL of each laccase preparation was applied to the gelatine-based hydrogel matrix containing ABTS (1.5 mg/mL), and the progress of the change in gel colour from transparent to green-blue was monitored in time.

#### 3.2.5. Determination of Storage Stability of 2,2′-Azino-bis(3-ethylbenzothiazoline-6-sulfonate) Sodium Salt

The ABTS is known to auto-oxidise, therefore its stability in native and immobilised form was verified after storage of the samples on the well plate at 4 °C for a specified time (0–20 days). The result for each ABTS formulation (solution or entrapped in hydrogel) stored for a particular time was compared with the results determined on day 0 (control sample).

#### 3.2.6. Determination of Storage Stability of the Hydrogel-Based Test

The stability of gelatine hydrogel matrices containing ABTS was verified by performing a detection test for a given concentration of laccase from *Trametes versicolor* by using matrices that were previously stored at 4 °C for a specified time (0–30 days) in closed well plates. The result for each hydrogel matrix stored for a particular time was compared with the results determined on day 0 (control sample). For this purpose, the intensity of the colour change after 30 min was compared with that at the start of the laccase detection test. The hydrogel assay was classified as stable under storage conditions if the effect of the change in gel colour from transparent to green-blue determined 30 min after the application of the enzyme solution at the same concentration (100 µg/mL) was similar to that obtained for the control test performed on day 0.

#### 3.2.7. Hydrogel-Based Assay for Colourimetric Screening a Substrate Specificity of Laccase

The test for screening the substrate specificity of laccase was based on the monitoring of the colour change of the solution potentially containing a substrate that was dropped on the gelatine matrix containing immobilised laccase (200 µg/mL). The appearance of the colour indicates that laccase effectively converts the given substrate to the colourful product. The intensity of the colour enables us to estimate the affinity strength of the laccase for the given substrate. It should be noted that this test is limited to substrates converted by the enzyme to colourful products. In our study, ABTS, guaiacol, and syringaldazine (SNG) were used as substrates that give green-blue, red-brown, and pink products, respectively. The ABTS and guaiacol were used as buffer solutions in the concentration range of 0.5–10 mg/mL. Syringaldazine was used as a methanolic solution in a lower concentration range (0.05–1.0 mg/mL) due to the poor solubility in water. The test was performed in a microlitre well plate filled with 200 µL hydrogel containing immobilised laccase by applying 100 µL of the tested solution potentially containing the substrate on the hydrogel surface and monitoring the appearance of the colour or lack thereof.

#### 3.2.8. Hydrogel-Based Assay for Colourimetric Screening Potential Inhibitors of Laccase

The test for screening potential inhibitors of laccase was based on the monitoring of the colour change of the ABTS solution potentially containing an inhibitor that was dropped on the gelatine matrix containing immobilised laccase. In the presented studies, sodium azide (NaN_3_) was used as a model inhibitor. The appearance of the deep green-blue colour indicated that laccase effectively converted the ABTS (1.5 mg/mL); however, the lower intensity of the colour or lack thereof was observed for solutions consisting of ABTS (1.5 mg/mL) and sodium azide at different concentrations (range of indicate the presence of inhibitor) in the analysed samples. The test was performed on a microlitre well plate. One hundred microliters of ABTS solution potentially containing the inhibitor was applied on the surface of a hydrogel with the immobilised laccase (200 µL). The differences in colour appearance between the solution potentially containing laccase inhibitor and control solution of ABTS itself were observed.

#### 3.2.9. Microorganism and Cultivation Conditions

A white-rot fungus *Cerrena unicolor* (Bull.ex.Fr.) Murr, strain no. 139 originated from the culture collection of the Department of Biochemistry, University of Lublin (Poland). The stock culture was maintained on potato dextrose agar at 4 °C and periodically transferred to a fresh medium. The fungus cultivation and laccase production were monitored for 16 days according to [2,88] with changes. On the fourth day, the culture was induced with pyrogallol dissolved in methanol to a final concentration of 10 µM [89]. The changes in pH, substrate, biomass, protein concentration, and laccase activity were determined by analysing the cultivation medium in a single flask corresponding to one day of cultivation. A detailed description of the culture cultivation can be found in Supporting Materials.

#### 3.2.10. Analytical Procedures

The mass of mycelium was determined by its separation from the cultivation medium through a paper filter, washing with distilled water, and drying at 85 °C to a constant mass. In the filtrate, pH, glucose, protein content, and laccase activity were determined. The pH measurements were performed with Crison Basic 20 pH-meter and a Crison 52 09 pH electrode at room temperature. The glucose amount was determined using the enzymatic test kit according to the procedure provided by a supplier (Biomaxima) and glucose as a standard. Protein content was determined with Lowry’s method [87] and albumin serum bovine as a standard. The analytical measurements were performed in triplicate with a standard deviation (SD) of less than 5%.

#### 3.2.11. Determination of Laccase Catalytic Activity

Laccase activity was determined based on the spectrophotometric measurements (λ = 420 nm, Shimadzu UV-1800) of the oxidation reaction rate using 1.5 mg/mL ABTS as a substrate in 0.1 M citrate-phosphate buffer (pH 5.2, 25 °C). The proportion of the enzyme to the substrate was 1:2 (*v*/*v*). The specific activity unit (U/mg) was defined as the amount of the enzyme (1 mg) that oxidises ABTS to the 1 µmole of the product (ε_420_ = 36 000 M^−1^cm^−1^) per minute at 25 °C.

## 4. Conclusions

In this article, we have reported the preparation and application of a quick and effective colourimetric test based on a hydrogel matrix for the determination of enzyme catalytic properties. The assay components were the laccase from *Trametes versicolor*, its substrate ABTS, and a gelatine hydrogel. We presented two approaches for using this test. The first concept concerned a hydrogel matrix with an immobilised substrate (ABTS) for application in the monitoring of laccase production in microbiological culture by detecting its oxidation activity. The second approach used immobilised laccase in the hydrogel for the determination of its substrate specificity for ABTS, syringaldazine, and guaiacol as well as evaluation of the inhibitor influence on enzyme activity with NaN_3_ as an example. The work was additionally supported by giving the characteristic parameters for the test, namely the detection sensitivity of the enzyme amount and pH range of examined samples, storage stability, and repeatability of the visual response. Despite the numerous advantages of this colourimetric test, there are some key requirements for its application: (i) the enzyme should retain its catalytic properties after immobilisation in the hydrogel matrix, (ii) the reaction product should be visually detectable, and (iii) the hydrogel matrix-substrate system should maintain a high level of enzyme detection for several weeks during storage at 4 °C.

We would like to emphasise that the examples given in this paper do not exhaust the application potential of the colourimetric test considered in the case of laccase. Other possible usages are studies of decolourisation processes, detoxification of wastewater from the textile industry, and detection of polyphenols or amines. The proposed well plate assay can be also prepared in such a way that it enables the simultaneous detection of enzymes with different biocatalytic properties. In particular, this property can be very useful for the selective screening of given biocatalysts in complex mixtures of microbial culture fluids. These examples offer a further research challenge for the development of new effective colourimetric tests based on different enzymes, substrates, and hydrogel matrices.

## Data Availability

Not applicable.

## Supplementary Materials

The supporting information can be downloaded at: https://www.mdpi.com/article/10.3390/ijms24010033/s1.
