# Supplementary material for: Colourimetric Plate Assays Based on Functionalized Gelatine Hydrogel Useful for Various Screening Purposes in Enzymology
Source: Int J Mol Sci. 2022 Dec 20;24(1):33. doi: 10.3390/ijms24010033 (PMC9819853; doi:10.3390/ijms24010033)
Supplement: Supplementary file 1 [file ijms-24-00033-s001.zip › ijms-2104735-supplementary.pdf]

SUPPORTING MATERIALS

Colourimetric plate assays based on functionalized gelatine hydrogels useful for various screening purposes in enzymology

Karolina Labus * and Halina Maniak

Department of Micro, Nano and Bioprocess Engineering, Faculty of Chemistry, Wroclaw University of Science and Technology, Wybrzeże Wyspiańskiego 27, Wrocław 50-370, Poland (

* Correspondence:
Tel.: +48-71-320-3314; fax: +48-71-328-1318// e-mail: karolina.labus@pwr.edu.pl (K.L.);

1. Conditions of *Cerrena unicolor* cultivation

A white-rot fungus *Cerrena unicolor* (Bull.ex.Fr.) Murr, strain no. 139 originated from the culture collection of the Department of Biochemistry, University of Lublin (Poland). The stock culture was maintained on potato dextrose agar at 4°C and periodically transferred to a fresh medium. The fungus cultivation and laccase production were performed according to [1] with changes. The culture medium (CM) included: glucose 12 g, L-asparagine 2.5 g, D,L-phenylalanine 0.15 g, adenine 0.027 g, KH_2PO_4 1.0 g, $Na_2HPO_4{\times}12H_2O$ 0.1 g, $MgSO_4{\times}7H_2O$ 0.5 g, and 10 mL of microelements' solution: $CaCl_2$ 0.01 g, $FeSO_4{\times}7H_2O$ 0.01 g, $MnSO_4{\times}4H_2O$ 0.001 g, $ZnCl_2$ 0.001 g, $CuSO_4{\times}5H_2O$ 0.002 g, and thiamine 50 µg, then the solution was replenished with distilled water up to 1 L. After sterilisation, the pH of the medium was 5.2. An inoculum of *C. unicolor* was prepared by harvesting mycelium with an inoculation loop from a six-day culture and directly transferring it to a 500 mL flask containing 100 mL of sterile CM. The microorganism pre-grew for 6 days on a rotary shaker (120 rpm) at a constant temperature of 24 °C. Consequently, a portion of 20 colonies of the *C. unicolor* with an average dry mass of 20±1.0 mg was transferred into 250 mL flasks containing 50 mL of sterile CM. The *C. unicolor* culture was growing for 16 days with a similar agitation and temperature regime. On the fourth day, fungal culture was induced with pyrogallol dissolved in methanol at a final concentration of 10 µM [2]. The concentration of alcohol in the culture medium was 0.4% (*v*/*v*). Changes in pH, substrate, biomass, protein concentration and laccase activity were determined by analysing the cultivation medium in a single flask corresponding to one day of cultivation.

2. Determination of bioprocess parameters

The yield factors for the applied growth conditions were calculated based on the following formulas:

$$Y_{\frac{X}{S}} = \frac{X_t - X_0}{S_0 - S_t} \qquad \text{(S1)}$$

$$Y_{\frac{P}{S}} = \frac{P_t - P_0}{S_0 - S_t} \qquad \text{(S2)}$$

Where, $Y_{X/S}$ (S1) stands for the yield coefficient of fungal biomass and $Y_{P/S}$ (S2) stands for the yield coefficient of the product (protein); X, S, and P correspond to the masses of biomass, substrate and protein (product), respectively; the symbols t and 0 in the subscript stand for the cultivation time, for which the masses of biomass, glucose and protein were used for calculations. A specific growth rate is determined from the logarithmic growth phase of a microorganism by plotting the relationship lnX= f(t).

3. Description of the *Cerrena unicolor* culture

The results of *Cerrena unicolor* cultivation are presented in **Table S1** (data correspond to **Figure 6** in the article). The monitoring of changes in biomass (X), glucose (S), product (P, protein), laccase activity (U/mg) and pH was carried out for 16 days. To avoid discrepancies due to water evaporation during the

experiment, the values of X, S, and P were expressed in units of mass [g]. The fungus was cultivated in batch mode, starting from 0.67 g (12 g/L) of glucose content. Complete depletion of the carbon substrate occurred within six days with a steady increase biomass and protein to 0.23 g (5.4 g/L) and 0.20 g (4.7 g/L), respectively. Yield factors calculated for time from 0 (beginning of the process) and day 6 were found $Y_{X/S}$ = 0.42 and $Y_{P/S}$ = 0.39. Furthermore, the specific growth rate μ [1/day], was determined as 0.71 day^{-1} at an average glucose concentration of 9.8 g/L and determined for a period of 1-3 days of the culture. Under the conditions described, the culture medium maintained the pH value at 5.3-6.4 between days 1 and 5 and increased to a slightly alkaline pH of 8.1 to 8.7 for up to the end of the experiment. Analysis of the course of laccase-specific activity showed that there were two peaks of maximum activity in the culture between days 6 and 8 and on day 11.

Table S1. The results of fungal biomass, pH, glucose, protein and laccase-specific activity measurements in *Cerrena unicolor* cultivation in the medium with glucose as the sole carbon source.

Time	Biomass (X)	pH	Glucose (S)	Protein (P)	Specific activity
[day]	[g]	[-]	[g]	[g]	[U/mg·10^{-1}]
0	0.000	5.27	0.666	0.090	0.000
1	0.026	5.27	0.599	0.092	0.000
2	0.065	5.67	0.553	0.125	0.005
3	0.096	5.99	0.393	0.145	0.005
4	0.130	6.39	0.178	0.125	0.057
5	0.190	5.86	0.124	0.129	0.144
6	0.226	8.07	0	0.198	0.484
7	0.206	8.3	0	0.218	$–^{nd}$
8	0.190	8.76	0	0.222	0.487
9	0.185	8.66	0	0.239	0.358
10	0.165	8.61	0	0.247	0.304
11	0.177	8.38	0	0.237	0.398
12	0.177	8.88	0	0.257	$–^{nd}$
14	0.166	8.81	0	0.276	0.340
16	0.161	8.69	0	0.299	0.289

$–^{nd}$ – not determined

4. Description of gelatine hydrogel cross-linked with microbial transglutaminase (mTGase)

Gelatine hydrogel used in our study was prepared according to the procedure described in detail in the article subsection no. **3.2.1**. As a result of cross-linking with mTGase, durable three-dimensional material was obtained. In order to use a hydrogel matrix enriched with active compounds for the preparation of a colorimetric test kit, just after mixing the gelatine solution with TGase, the resulting mixture was placed in a 96-well plate in the amount of 200 μL to each well. In this way, a transparent support was obtained for simultaneous multi-sample screening (**Figure S1**).

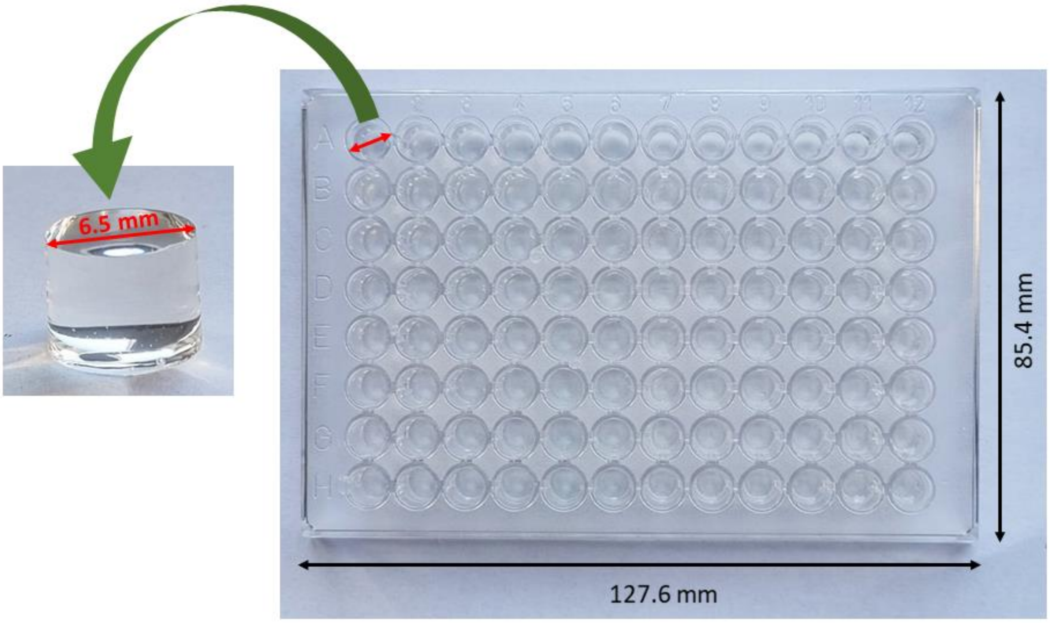

Figure S1. Gelatine hydrogel used for development of colourimetric test for various screening purposes in enzymology (left) and the 96-well plate used (right).

5. References

1. Fahraeus, G.; Reinhammar, B. Large scale production and purification of laccase from cultures of the fungus Polyporus versicolor and some properties of laccase A. *Acta Chem. Scand.* **1967**, *21*, 2367–2378.
2. Al-adhami, A.J.H.; Bryjak, J.; Greb-Markiewicz, B.; Peczynska-Czoch, W. Immobilization of wood-rotting fungi laccases on modified cellulose and acrylic carriers. *Process Biochem.* **2002**, *37*, 1387–1394.
